# The minimal clinically important difference of the control of allergic rhinitis and asthma test (CARAT): cross-cultural validation and relation with pollen counts

**DOI:** 10.1038/npjpcrm.2014.107

**Published:** 2015-01-08

**Authors:** Sander van der Leeuw, Thys van der Molen, PN Richard Dekhuijzen, Joao A Fonseca, Frederik A van Gemert, Roy Gerth van Wijk, Janwillem WH Kocks, Helma Oosterom, Roland A Riemersma, Ioanna G Tsiligianni, Letty A de Weger, Joanne NG Oude Elberink, Bertine MJ Flokstra-de Blok

**Affiliations:** 1 Department of General Practice, University of Groningen, University Medical Center Groningen, Groningen, The Netherlands; 2 GRIAC Research Institute, University of Groningen, University Medical Center Groningen, Groningen, The Netherlands; 3 Department of Pulmonology, University Medical Center St Radboud, Nijmegen, The Netherlands; 4 Department of Health Information and Decision Sciences, Faculdade de Medicina da Universidade do Porto, Porto, Portugal; 5 Department of Internal Medicine—Allergology, Erasmus Medical Center, Rotterdam, The Netherlands; 6 Department of Pulmonology, Leiden University Medical Center, Leiden, The Netherlands; 7 Department of Allergology, University of Groningen, University Medical Center Groningen, Groningen, The Netherlands

## Abstract

**Background:**

The Control of Allergic Rhinitis and Asthma Test (CARAT) monitors control of asthma and allergic rhinitis.

**Aims:**

To determine the CARAT’s minimal clinically important difference (MCID) and to evaluate the psychometric properties of the Dutch CARAT.

**Methods:**

CARAT was applied in three measurements at 1-month intervals. Patients diagnosed with asthma and/or rhinitis were approached. MCID was evaluated using Global Rating of Change (GRC) and standard error of measurement (s.e.m.). Cronbach’s alpha was used to evaluate internal consistency. Spearman’s correlation coefficients were calculated between CARAT, the Asthma Control Questionnaire (ACQ5) and the Visual Analog Scale (VAS) on airway symptoms to determine construct and longitudinal validity. Test–retest reliability was evaluated with intra-class correlation coefficient (ICC). Changes in pollen counts were compared with delta CARAT and ACQ5 scores.

**Results:**

A total of 92 patients were included. The MCID of the CARAT was 3.50 based on GRC scores; the s.e.m. was 2.83. Cronbach’s alpha was 0.82. Correlation coefficients between CARAT and ACQ5 and VAS questions ranged from 0.64 to 0.76 (*P*<0.01). Longitudinally, correlation coefficients between delta CARAT scores and delta ACQ5 and VAS scores ranged from 0.41 to 0.67 (*P*<0.01). Test–retest reliability showed an ICC of 0.81 (*P*<0.01) and 0.80 (*P*<0.01). Correlations with pollen counts were higher for CARAT than for ACQ5.

**Conclusions:**

This is the first investigation of the MCID of the CARAT. The CARAT uses a whole-point scale, which suggests that the MCID is 4 points. The CARAT is a valid and reliable tool that is also applicable in the Dutch population.

## Introduction

Asthma and allergic rhinitis are common diseases that have a negative influence on social life, school performance and work productivity.^1^ Epidemiologic studies have shown that asthma and allergic rhinitis frequently co-exist; i.e., 70–90% of patients with asthma also have allergic rhinitis and 40–50% of patients with allergic rhinitis also have asthma.^2,3^ Furthermore, there is a probable association between the severity of asthma and allergic rhinitis.^2–5^

Most patients with asthma and/or allergic rhinitis are treated by general practitioners. Given the variation in patients, symptoms and clinical presentation, general practitioners face uncertainty regarding asthma and allergic rhinitis management.^6,7^ Although the majority of asthma patients also suffer from allergic rhinitis, generally used questionnaires for asthma disease control do not take into account the impact of allergic rhinitis.^8–11^ In addition, general practitioners are often not aware of allergic rhinitis symptoms in their asthma patients, although the allergic rhinitis symptoms might have a large impact on their asthma control.^12^This results in a large number of patients who do not receive appropriate care despite the availability of effective treatment options.^11^

The ARIA guidelines recommend optimal control of both asthma and allergic rhinitis airway disease as the primary goal of their treatment.^1,13^ A combined approach of upper and lower airway disease management is a key issue that has been extensively proposed.^1,13–15^ To assess the effects of treatment on the control, validated questionnaires have been identified as key instruments.^16–18^

The Control of Allergic Rhinitis and Asthma Test (CARAT) questionnaire was created and validated to measure disease control of both asthma and allergic rhinitis.^19,20^ However, the minimal clinically important difference (MCID), a vital measure for the interpretation of CARAT scores,^21^ is unknown. Moreover, the psychometric properties of the CARAT have not yet been tested outside the country of development. The objective of this study was to establish the MCID of the CARAT. Additional objectives were to evaluate the internal consistency, cross-sectional validity, longitudinal validity and test–retest reliability for the Dutch CARAT version. Furthermore, the sensitivity of the CARAT in relation to the influence of pollen counts was investigated.

## Materials and Methods

### Study design and procedure

The longitudinal study consisted of three measurements (T1, T2 and T3) with 1-month intervals during the period May through August 2012. At each time point, the questionnaire packages included CARAT, an asthma control questionnaire (ACQ5), three visual analog scales (VAS) on pulmonary symptoms, and a question concerning medication use during the preceding month. The questionnaire packages at T2 and T3 also included a global rating of change (GRC) question. The questionnaires were distributed to the patients by mail with an accompanying letter from both their clinician and the researcher, explaining the objectives of the study, along with an informed consent form. Participants were requested to return the completed questionnaires and informed consent form in a prepaid envelope. Reminders were sent once, in case a participant had not returned the questionnaire within 1 week after the aimed date.

### Participants

Patients were recruited from the outpatient clinics of the departments of allergy or pulmonary diseases of the university hospitals in Groningen, Rotterdam and Nijmegen. In addition, patients were recruited from primary care practices in Groningen, Appingedam and Harlingen. Patients aged 18–70 years with a physician diagnosis of asthma and/or allergic rhinitis were asked to participate. Patients with insufficient command of the Dutch language and those diagnosed with dementia were excluded from the study. At each centre, baseline characteristics of the patients were obtained from patient files. These characteristics included age, sex, diagnosis, age of onset, respiratory co-morbidity, type of allergen and, where available, skin prick test, specific IgE and/or lung function. Informed consent was obtained from all patients. The local Medical Ethical Review Commission deemed that permission from the commission was not required (METc 2012.096).

### Outcome measures

#### CARAT

The CARAT consists of 10 questions scored on a 4-point Likert scale with a recall period of 4 weeks.^20^ Seven questions relate to the frequency of airway symptoms, four of which focus on upper airway symptoms and three focus on lower airway symptoms. The other three questions deal with sleep impairment, activity limitations and the need for higher doses of medication. The total score is calculated by summing up the scores of all 10 questions, resulting in a range of 0–30 points, with a higher score representing better control. The CARAT consists of two domains: allergic rhinitis (question no. 1–4) and asthma (question no. 5–10).^20^ The CARAT was originally developed and validated in Portugal and translated into Dutch following international recommendations. These comprise repetitive rounds of forward translation, backward translation, comparison of back translation with original until consensus is obtained by the expert panel and testing in patients in terms of comprehension.^22^

#### ACQ5

The asthma control questionnaire (ACQ5) consists of five questions that are scored on a 7-point Likert scale with a recall period of 1 week. The total ACQ5 score is the mean score of all questions (ranging from 0 to 6), a lower score representing better control. The ACQ5 has been shown to be reliable (intra-class correlation coefficient (ICC)=0.90, *P*<0.0001) and has strong evaluative properties for the measurement of asthma control.^16^ It has also been shown to have good discriminative properties to distinguish patients who have well-controlled asthma (score ⩽0.75 points) from those with uncontrolled asthma (score ⩾1.5 points).^23^

#### VAS

Three visual analog scales were used to assess all airway symptoms (VAS-all), lower airway symptoms (VAS-low) and upper airway symptoms (VAS-up). Participants were asked to mark the position on a 10-centimetre line corresponding to the amount of symptoms they experienced in the preceding week.^24^

#### GRC

At T2 and T3, a global rating of change (GRC) question with a 15-point scale was used to monitor the participants’ subjective experience of change in symptoms of asthma and allergic rhinitis, compared with the previous measurement. The score range of this question was from −7 (extremely worse) through 0 (no change) to 7 (extremely better). The GRC question was used for the determination of the MCID of the CARAT.^25^

#### Pollen counts

During the study, daily pollen-specific counts were provided by the Leiden University Medical Center. Grass and birch pollen counts were used for the analysis because they are the major cause of pollen-induced symptoms in Northern Europe.^26^

### Statistical analyses

Statistical analyses were performed using SPSS 19.

The MCID of the CARAT was established using both an anchor-based and a distribution-based method. For the anchor-based method, the GRC scores at T2 were used. This GRC measurement represents the difference in symptoms between T1 and T2. Patients were divided into four categories on the basis of GRC scores: no difference (−1, 0, 1), minimal difference (−3,−2, 2, 3), moderate difference (−5, −4, 4, 5) and large difference (−7, −6, 6, 7). For each category, the mean difference in CARAT score between T1 and T2 was calculated. The outcome of the GRC category minimal difference was considered as the MCID.^25^ For the distribution-based method, the standard error of measurement (s.e.m.) was calculated using the CARAT scores at T1 and used as a threshold to further establish the MCID.

The internal consistency of the CARAT was evaluated by calculating Cronbach’s alpha. A Cronbach’s alpha of at least 0.70 is required for the comparison of groups of patients.^27^

The cross-sectional construct validity of the CARAT was evaluated by calculating Spearman’s correlation coefficients for CARAT (total and domains scores) with ACQ5 and VAS scores (VAS-all, VAS-up and VAS-low). *A priori* expectations were based on the Portuguese version of the CARAT, which showed correlation coefficients ranging from 0.6 to 0.8 with the ACQ5 and VAS scores.^20^ The CARAT domain allergic rhinitis was expected to correlate best with VAS-up and the CARAT domain asthma was expected to correlate best with VAS-low.

The longitudinal validity of the CARAT was evaluated by calculating Spearman’s correlation coefficients for delta scores of the CARAT (total and domains scores) with delta scores of the ACQ5 and VAS scores. Delta scores were calculated as T2 minus T1 and T3 minus T2. *A priori* expectations were based on the Portuguese version of the CARAT, which showed longitudinal correlation coefficients ranging from 0.4 to 0.6 with the ACQ5 and VAS scores.^21^

The test–retest reliability of the CARAT was evaluated by calculating ICC of CARAT scores for the first interval (T1, T2) and second (T2, T3) interval. Only patients in the ‘no difference’ category based on the GRC scores measured at T2 and T3 were included in these analyses.

The discriminative properties of the CARAT were investigated by dividing the patients into two groups on the basis of ACQ5 scores at T1: (1) patients with ACQ5 score <1.5 (well and partly controlled) and (2) patients with ACQ5 score ⩾1.5 (uncontrolled).^23^ The mean CARAT score for both the groups was compared using an independent samples *t*-test. The *a priori* expectation was to find a significantly lower mean CARAT score for group 2 compared with group 1.

To investigate the sensitivity of the CARAT to the influence of pollen counts, patients with a history of clinical reactivity to pollen (grass and/or birch) were selected. Depending on the date of completing the questionnaire package, a mean pollen count over the previous 4 weeks was calculated for each patient at each measurement point (T1, T2 and T3). Pearson’s correlation coefficients were calculated between delta pollen counts and delta CARAT scores, as well as delta ACQ5 scores. A higher correlation with pollen counts was expected for CARAT scores than for ACQ5 scores.

## Results

Of the 176 approached patients, 92 patients completed T1 (response rate 53%). Response rates of T2 (89%) and T3 (88%) were much higher. An equal number of patients were recruited from primary and secondary care (Table 1).

The mean score of the CARAT among all patients for T1 was 19.4, with a standard deviation of 6.8 (Table 2). There was no floor effect as no patients scored the minimum score of 0 (worst control) but there was a small ceiling effect with four patients scoring the maximum score of 30 (best control).

### Minimal clinically important difference

Mean CARAT scores for each GRC category are listed in Table 3. The MCID for total CARAT scores, derived from the GRQ category ‘minimal difference’, is 3.50. CARAT scores showed an s.e.m. of 2.83.

### Internal consistency

The Cronbach’s alpha for the total CARAT questionnaire was 0.82 on T1, with an alpha of 0.81 for domain allergic rhinitis and 0.77 for domain asthma. Cronbach’s alpha for the total CARAT questionnaire was 0.86 on T2 and 0.83 on T3.

### Construct validity

Correlation coefficients of CARAT total and domain scores with ACQ5 and VAS scores are shown in Table 4. All *a priori* expected best correlations were met.

### Longitudinal validity

Correlation coefficients between delta scores for the CARAT and delta scores for the ACQ5 and VAS scores are shown in Table 5. *A priori* expected correlations were confirmed.

### Test–retest reliability

Test–retest reliability of the CARAT was confirmed by an ICC of 0.81 (*P*<0.01) for T1–T2 (*n*=44) and 0.80 (*P*<0.01) for T2–T3 (*n*=31).

### Discriminative properties

On T1, the group with ‘well and partly controlled’ asthma according to the ACQ5 (*n*=70) had a mean CARAT score of 21.43 (s.d. 5.85). The group with ‘uncontrolled’ asthma according to the ACQ5 (*n*=22) showed a mean CARAT score of 12.77 (s.d. 5.32). The difference in means between both groups was significant (*P*<0.001).

### Pollen counts

Correlation coefficients of delta CARAT and ACQ5 scores with delta pollen counts are shown in Table 6. Correlations with pollen counts were higher for the CARAT than for ACQ5.

## Discussion

### Main findings

This is the first study that determines the MCID of the CARAT and indicates that this stands at 3.5 points based on GRC analysis. As the CARAT score is a whole-point scale, this outcome suggests that a change in score of 4 points or more from baseline indicates the smallest change in control of asthma and allergic rhinitis as measured by the CARAT that can be considered as clinically significant. The determination of MCID is important for interpreting CARAT scores and, therefore, is a vital step for implementation in clinical practice.^22^ This is also the first study to investigate the psychometric properties of the CARAT in another country than the country of origin (Portugal) and we have found that the Dutch CARAT is a valid tool with good internal consistency and discriminative properties.

### Strengths and limitations of this study

One of the strengths of this study is the determination of the MCID by using both an anchor-based method and a distribution-based method. In addition, both patients from primary and secondary care were included in this study. This is also the first study reporting on the psychometric properties of the CARAT in another country than the country of origin showing that it is valid and reliable. Further, this was the first study in which CARAT was administered by mail. A limitation of this study is the attrition rate at T2 and T3. For each measuring moment, the number of patients reduces by 10. For this reason, the main focus in the interpretation of the results lies with T1. The sample size for each measurement (T1, T2 and T3) is considered sufficient, as a number of 50–100 patients is usually what is needed in questionnaire validation studies.^28^

Another limitation is that pollen counts were measured in Leiden, whereas participants were recruited from Rotterdam, Nijmegen, Groningen and Friesland. Although the maximum distance between the pollen station and the centres is only 200 km, local fluctuations of pollen counts may have occurred.

A final limitation of the study is the incomplete descriptive baseline characteristics concerning lung function, specific IgE and skin prick tests, especially for primary care patients. However, these are not standard clinical investigations in primary care and all available data were included.

### Interpretation of findings in relation to previously published work

The MCID of the CARAT was evaluated using both an anchor-based method (GRC) and a distribution-based method (s.e.m.). The rationale for using the s.e.m. as a tool to further confirm the MCID is that when a change is smaller than the s.e.m., it is probably a measurement error rather than a true change.^29^ However, this does not indicate whether the magnitude of change is important for patients as perceived by patients.^30^ Therefore, from the clinical point of view, the anchor-based method using GRC would be preferred to establish MCID with the s.e.m. as an establishment threshold. The establishment of the MCID of the CARAT has been proposed as an important step for meeting COSMIN requirements.^28^ With the CARAT meeting 9 out of 10 criteria so far, this has been marked as a highly prioritised goal.^22^

The Dutch CARAT (total and domains) showed satisfactory internal consistency, which was comparable to that of the Portuguese CARAT study.^20^ With regard to the construct validity, all *a priori* expectations were met. As expected, the CARAT asthma domain showed good correlation with ACQ5, which measures asthma control, and the CARAT allergic rhinitis domain showed good correlation with VAS-up, which covers upper airway symptoms. Comparisons of the CARAT domains with the lower and upper airway domains vice versa showed lower correlation coefficients throughout. Thus, the separate CARAT domains measure the supposed construct. These results were similar to findings for the Portuguese version of the CARAT,^20^ underlining good cross-sectional validity of the CARAT.

The Dutch CARAT showed satisfactory longitudinal validity as well. Correlation coefficients between delta scores of the CARAT and delta scores of the ACQ5 and VAS questions were reasonably high. These results underline findings in the Portuguese CARAT study, which showed similar longitudinal results. The same was true for the test–retest reliability of the Dutch CARAT.^21^ The Dutch CARAT is also shown to be able to distinguish patients on the basis of ACQ5 cut-off scores. Therefore, discriminative properties of the Dutch CARAT are good when it comes to distinguishing ‘well controlled and partly controlled’ patients from ‘uncontrolled’ patients on the basis of ACQ5 scores.^23^

As expected, higher correlation coefficients with pollen counts were found for CARAT than for ACQ5. Although the correlation coefficients were not persuasively high, they suggest an association between delta pollen counts and delta CARAT score. Moreover, high correlations were not expected, given the fact that the majority of patients in this study were well controlled and, therefore, less likely to be sensitive to rising pollen counts. Furthermore, patients suffering from allergic rhinitis have been shown to have more severe symptoms in the early flowering season in relation to peaks in pollen counts, when compared with peaks later in the same season.^31^ This can be explained by the fact that patients allergic to pollen may have a potential to downregulate their allergic response after repeated allergen exposure.^32^ This could also explain the lower correlation coefficients that were found in the second interval (T2–T3).

### Implications for further research, policy and practice

Validated questionnaires have been suggested as key instruments for the evaluation of all airway symptoms.^16–18^ The Dutch CARAT is therefore a valuable addition to existing questionnaires that generally evaluate only lower airway symptoms.^8–11^ Accordingly, the CARAT is a useful tool in the Netherlands for facilitating optimal control of both asthma and allergic rhinitis simultaneously. This has been extensively proposed as a future goal to be achieved.^1,12^

### Conclusions

This is the first study evaluating the MCID of the CARAT, suggesting an MCID at 4 points. The determination of MCID is highly important to be able to interpret CARAT scores and is a vital step for implementation in clinical practice. The CARAT is a valid and reliable tool for monitoring asthma and allergic rhinitis symptoms simultaneously, which has been extensively proposed by ARIA guidelines. Moreover, CARAT scores seem to be more sensitive to changes in pollen counts when compared with ACQ5 scores.

## Figures and Tables

**Table 1:** Patient characteristics

*Characteristics*	*Primary care*	*Secondary care*
Patients, *n*	46	46
Age in years, mean (s.d.)	46.6 (12.7)	41.3 (14.2)
		
*Sex,* n *(%)*		
Male	19 (41.3)	11 (23.4)
Female	27 (58.7)	35 (76.6)
		
*Diagnosis,* n *(%)*		
Asthma	26 (56.5)	26 (55.3)
Rhinitis	31 (67.4)	46 (100.0)
		
*Age of onset in years, mean (s.d.)*		
Asthma	37.0 (15.1)	19.8 (14.5)
Rhinitis	32.7 (11.1)	19.4 (10.5)
Respiratory co-morbidity^a^ *n* (%)	4 (8.7)	11 (23.9)
		
*Type of allergen* b, n *(%)*		
Not allergic	12 (26.1)	1 (2.2)
Pollen	18 (39.1)	39 (84.8)
Non-pollen allergen	8 (17.4)	5 (10.9)
Unknown	8 (17.4)	1 (2.2)
ACQ5 score, mean (s.d.)	1.1 (1.2)	0.9 (0.9)
Skin prick test, *n* (%)	1 (2.2)	15 (32.6)
Specific IgE, *n* (%)	2 (4.4)	30 (65.2)
Lung function, *n* (%)	41 (89.1)	33 (71.7)
% predicted FEV1, mean (s.d.)	102.3 (14.4)	98.7 (15.4)
		
*Medication use* c, n *(%)*		
Antihistamines		
Local	1 (2.2)	7 (15.2)
Systemic	7 (15.2)	21 (45.7)
Corticosteroids		
Nasal	10 (21.7)	32 (69.6)
Pulmonal	19 (41.3)	18 (39.1)
Systemic	2 (4.3)	3 (6.5)
B-sympathicomimetics		
Short-acting	12 (26.1)	29 (63.0)
Long-acting	13 (28.3)	10 (21.7)
Leukotrien antagonists	4 (8.7)	8 (17.4)
Immunotherapy	0 (0.0)	3 (6.5)
Decongestives	3 (6.5)	0 (0.0)

Abbreviations: ACQ, Asthma Control Questionnaire; FEV1, forced expiratory volume in 1 second.

a Chronic obstructive pulmonary disease exclusively.

b Based on history. Data for skin prick tests and specific IgE could not be described because of different outcome measures at each centre.

c Medication as taken by patients.

**Table 2:** Mean CARAT scores

*CARAT scores*	*Mean (s.d.)*	n
CARAT T1	19.36 (6.80)	92
CARAT T1 ♀	18.63 (6.59)	63
CARAT T1 ♂	20.93 (7.11)	29
		
*CARAT T1 age percentiles*		
<33	17.87 (7.07)	23
34–43	17.38 (7.64)	23
44–53	19.38 (7.04)	23
>54	22.24 (4.88)	23
CARAT T2	19.99 (7.13)	82
CARAT T3	21.67 (6.51)	72

Abbreviation: CARAT, Control of Allergic Rhinitis and Asthma Test.

**Table 3:** Minimal clinically important difference

*Difference category*	*GRC*	*Difference CARAT score mean (s.d.)*
None (*n*=44)	−1, 0, 1	3.00 (2.79)
Minimal (*n*=16)	−3, −2, 2, 3	**3.50 (2.78)**
Moderate (*n*=15)	−5, −4, 4, −5	7.07 (4.08)
Large (*n*=7)	−7, −6, 6, 7	5.57 (6.40)

The MCID (minimal clinically important difference) is shown in bold.

Abbreviations: CARAT, Control of Allergic Rhinitis and Asthma Test; GRC, Global Rating of Change (−7 through 7).

**Table 4:** Spearman correlations construct validity

*T1*	*ACQ*	*VAS-all*	*VAS-low*	*VAS-up*	*VAS-low+up*
CARAT total score	−0.66	**−0.69**	−0.62	−0.64	**−0.76**
CARAT domain allergic rhinitis	−0.41	−0.47	−0.41	**−0.70**	−0.66
CARAT domain asthma	**−0.70**	−0.68	**−0.64**	−0.44	−0.71

Abbreviations: ACQ, Asthma Control Questionnaire; CARAT, Control of Allergic Rhinitis and Asthma Test; VAS-all, Visual Analog Scale-all airway symptoms; VAS-low, Visual Analog Scale-lower airway symptoms; VAS-up, Visual Analog Scale-upper airway symptoms; VAS-low+up, Sum of VAS-low and VAS-up score.

All correlations were statistically significant (*P*<0.01). The *a priori* expected best correlations are shown in bold.

**Table 5:** Spearman correlations longitudinal validity

	*Period 1 (T1-T2)*	*Period 2 (T2-T3)*
*CARAT total scores*		
CARAT—ACQ	0.45	0.40
CARAT—VAS-all	0.61	0.45
CARAT—VAS-low+up	0.67	0.40
		
*CARAT domains*		
Domain allergic rhinitis—VAS-up	0.55	0.36
Domain asthma—VAS-low	0.45	0.29
Domain asthma—ACQ	0.41	0.41

All correlations were statistically significant (*P*<0.01). Period 1 represents the 4-week period between T1 and T2, period 2 represents the 4-week period between T2 and T3.

Abbreviations: ACQ, Asthma Control Questionnaire; CARAT, Control of Allergic Rhinitis and Asthma Test; VAS-all, Visual Analog Scale-all airway symptoms; VAS-low, Visual Analog Scale-lower airway symptoms; VAS-up, Visual Analog Scale-upper airway symptoms; VAS-low+up, Sum of VAS-low and VAS-up score.

**Table 6:** Longitudinal association of delta CARAT and ACQ scores with delta pollen counts in patients with a history of clinical reactivity to pollen

	*Delta pollen counts birch/grass*	
	*Period 1 (*n*=49)*	*Period 2 (*n*=41)*
Delta CARAT score	0.32	0.11
Delta ACQ score	0.10	0.06

All Pearson correlations were significant (*P*<0.05). Period 1 represents the 4-week period between T1 and T2, period 2 represents the 4-week period between T2 and T3.

Abbreviations: ACQ, Asthma Control Questionnaire; CARAT, Control of Allergic Rhinitis and Asthma Test.
